# Iron Homeostasis as a Mediator Linking Central Obesity with MASLD and Primary Liver Cancer: A Two-Step Mendelian Randomization Study

**DOI:** 10.3390/biomedicines13071641

**Published:** 2025-07-04

**Authors:** Yuping Zeng, Xia Wang, Shenlin Liao, Chuan Li, Jie Chen, He He

**Affiliations:** 1 Department of Laboratory Medicine, West China Hospital, Sichuan University, Chengdu 610041, China; 2 Sichuan Clinical Research Center for Laboratory Medicine, Chengdu 610041, China; 3 Clinical Laboratory Medicine Research Center, West China Hospital, Chengdu 610041, China; 4 Department of Blood Transfusion, West China Hospital, Sichuan University, Chengdu 610041, China; 5 Department of Thoracic Surgery, West China Hospital, Sichuan University, Chengdu 610041, China

**Keywords:** central obesity, ferritin, Mendelian randomization, causality

## Abstract

**Objectives**: This study aimed to explore the mediating effects of iron homeostasis biomarkers linking central obesity with metabolic dysfunction-associated steatotic liver disease (MASLD) and primary liver cancer (PLC) via Mendelian randomization (MR) analysis. **Methods**: Two-sample bidirectional MR, multivariable MR, and mediation analyses were used to investigate the causal associations among obesity-related traits, iron homeostasis biomarkers, MASLD, and PLC. For the discovery and replication analyses, GWAS summary data for iron homeostasis biomarkers, MASLD, and PLC were extracted from two datasets, and the combined effects were pooled to corroborate the conclusions. **Results**: BMI and waist circumference were associated with a risk of MASLD in their combined effects (OR = 1.83, 95% CI = 1.33–2.52 for BMI; OR = 1.98, 95% CI = 1.63–2.41 for waist circumference). Waist circumference but not BMI had significant causal effects on the risk of PLC in the discovery dataset (OR = 1.71, 95% CI = 1.01–2.89 for BMI; OR = 2.72, 95% CI = 1.37–5.39 for waist circumference). In both of the iron homeostasis datasets, genetically predicted increased ferritin was associated with increased risk of MASLD by multivariable MR. We only observed that genetic liability to increased ferritin was associated with increased risk of PLC in iron homeostasis dataset 1 after adjusting for waist circumference. By two-step MR analysis, we found that genetic liability to ferritin mediated 3.34% (95% CI: 0.17–8.08%) of waist circumference effects on MASLD risk and 18.84% (95% CI: 3.01–40.51%) of its effects on PLC risk. **Conclusions**: Waist circumference and iron homeostasis biomarkers were causally associated with increased risks of MASLD and PLC. Central obesity may contribute to the development of MASLD and PLC by increasing ferritin levels.

## 1. Introduction

Obesity is a serious global health concern [1] with adverse effects including diabetes, metabolic syndrome, metabolic dysfunction-associated steatotic liver disease (MASLD), cardiovascular disease, carcinogenesis, and many others [2,3,4]. MASLD, previously known as non-alcoholic fatty liver disease (NAFLD) [5], encompasses a wide spectrum of diseases, ranging from simple steatosis to metabolic dysfunction-associated steatohepatitis (MASH), fibrosis, cirrhosis, and even end-stage hepatocellular carcinoma (HCC) [6]. MASH-related deaths are predicted to increase by 178% by 2030, making them the leading cause of liver-related mortality in the future; nevertheless, as of right now, there are few approved efficient treatment medications for MASLD [7]. Obesity is a prominent cause of MASLD, and patients could benefit from weight loss and bariatric–metabolic surgery [8]. Research has also reported that as obesity rates rise, the primary etiology of HCC is dynamically shifting from viral to metabolic liver diseases [9]. While the link between obesity and the development of MASLD and primary liver cancer (PLC) has been extensively studied, the underlying mechanisms remain incompletely understood.

Iron homeostasis is a possible mediator linking obesity with MASLD and PLC [10]. Iron, a redox-active metal, can induce oxidative stress, endoplasmic reticulum stress, inflammation, and dysfunction of the adipose tissue endocrine system [11]. Research has shown that hepatic iron deposition is strongly correlated with serum ferritin levels, and that iron homeostasis imbalance and ferroptosis are attributed to the pathogenesis of cardiometabolic and liver diseases [12,13]. However, some observational studies have shown that serum iron levels are decreased in MASLD and PLC patients [14,15]. Furthermore, elevated circulating levels of hepcidin and systemic iron insufficiency are prevalent in patients with obesity [16]. Therefore, iron homeostasis appears to be debatable in obesity and metabolic disorders. It is necessary to further investigate the associations among obesity, iron homeostasis biomarkers, and liver diseases.

Mendelian randomization (MR) [17] is an instrumental variables (IVs) analysis approach that mainly uses single nucleotide polymorphisms (SNPs) as IVs. The main principle is similar to that of randomized controlled trials; that is, SNPs are randomly assigned to offspring during meiosis and the populations are divided into high- or low-exposure groups [18]. Compared with traditional epidemiological studies, MR analysis can effectively avoid confounding factors and reverse causality [19]. Several MR studies have explored the causal relationships between iron homeostasis biomarkers and liver diseases [20,21,22,23]. To the best of our knowledge, few studies have examined the causal link among overall obesity, central obesity, MASLD, and PLC from a genetic perspective [24]. Moreover, whether obesity increases the risks of MASLD and PLC through the iron homeostasis pathway needs to be further studied.

To investigate the potential role of iron homeostasis biomarkers as mediators in the obesity–liver disease pathway, we conducted two-sample bidirectional MR, multivariable MR, and mediation analyses to elucidate the relationships among obesity-related traits, iron homeostasis biomarkers, MASLD, and PLC.

## 2. Materials and Methods

### 2.1. Study Design

**Figure 1:**
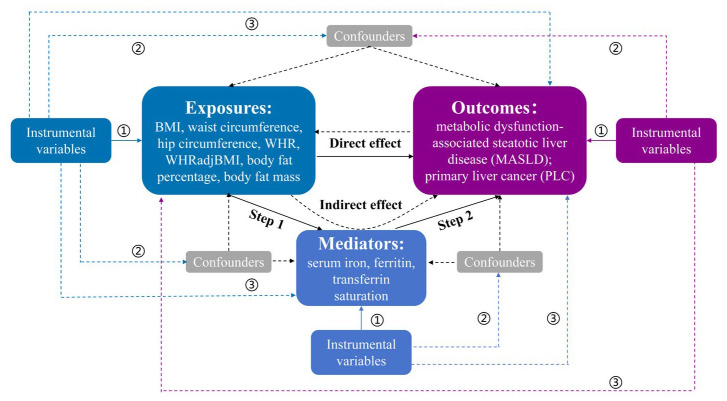
An overview of the study design. The Mendelian randomization (MR) analysis satisfied three core assumptions. ➀: instrumental variables (IVs) are significantly associated with exposures; ➁: IVs are not related to confounders; ➂: IVs influence the outcomes only though the exposures. WHR: waist–hip ratio; WHRadjBMI: WHR adjusted for BMI.

Figure 1 illustrates in detail the flowchart of the study design. First, a two-sample bidirectional MR analysis was conducted to estimate the causal effects of obesity-related traits (exposures) on MASLD and PLC risks (outcomes). After adjusting for obesity-related traits, multivariable MR analysis was subsequently used to estimate the associations between iron homeostasis biomarkers (mediators) and outcomes. Finally, a two-step MR analysis explored the mediating effects of iron homeostasis biomarkers linking obesity with MASLD and PLC. This study followed the Strengthening the Reporting of Observational Studies in Epidemiology using MR (STROBE-MR) guidelines [25].

### 2.2. Data Sources

We retrieved genetic data on obesity-related traits, including body mass index (BMI), waist circumference, and hip circumference from the Neale Lab Consortium (http://www.nealelab.is/uk-biobank/, accessed on 30 June 2025). The waist–hip ratio (WHR) and WHR adjusted for BMI (WHRadjBMI) were obtained from the Genetic Investigation of Anthropometric Traits (GIANT) consortium, which included 212,244 individuals in the genome-wide association studies (GWASs) meta-analyses of waist- and hip-circumference-related traits [26]. Body fat percentage and body fat mass were obtained from the MRC-IEU consortium (https://www.bristol.ac.uk/integrative-epidemiology/, accessed on 30 June 2025). Iron homeostasis biomarkers, including serum iron, ferritin, and transferrin saturation were retrieved from the GWAS meta-analysis of iron homeostasis consisting of 23,986 subjects (iron homeostasis dataset 1) [27]. For confirming our findings, another GWAS summary dataset of iron homeostasis biomarkers (iron homeostasis dataset 2) was obtained from https://www.decode.com/summarydata/ (accessed on 30 June 2025) [28].

For discovery and replication analysis, GWAS summary data for MASLD and PLC were taken from two datasets. Summary-level data on NAFLD were extracted from a GWAS including 8434 NAFLD cases and 770,180 controls [29], and from another GWAS including 4761 NAFLD cases and 373,227 controls [30]. The GWAS summary data for PLC included 304 cases for the discovery dataset (FinnGen consortium) and 379 cases for the replication dataset [31]. Moreover, biomarkers of liver injury and hepatic steatosis including alanine aminotransferase (ALT), aspartate aminotransferase (AST), gamma glutamyl transpeptidase (GGT), alkaline phosphatase (ALP), triglycerides, and fasting blood insulin were retrieved from the summary of GWAS statistics [31,32]. All GWAS summary data were extracted from the MR-Base database (https://www.mrbase.org, accessed on 30 June 2025) [33]. Detailed information on the GWASs of the data sources, including authors, PMID, and sample sizes is presented in Tables S1 and S2.

### 2.3. Genetic Instrument Selection

SNPs that showed strong associations with obesity-related traits (*p* < 5.0 × 10^−8^) were selected as candidate IVs. Given the limited number of significantly linked IVs for PLC and MASLD, genetic variations related to these exposures were selected at a genome-wide significance level of *p* < 1.0 × 10^−6^ in the reverse MR analysis. For multivariable MR (MVMR) analysis, IVs were selected at the genome-wide significance level of *p* < 5.0 × 10^−8^ to guarantee that there was a high correlation between the IVs and exposures. Clumping analysis was subsequently performed according to the criterion that the R^2^ of the linkage disequilibrium was less than 0.01 at a 10,000 kb window using the European population reference. In addition, palindromic SNPs with intermediate allele frequencies were excluded. Finally, these independent, genome-wide-significant, and nonpalindromic SNPs were included as IVs. Descriptive information on the SNPs used as IVs associated with obesity-related traits and iron homeostasis biomarkers is shown in Supplementary Data S1–S16.

### 2.4. Statistical Analysis

#### 2.4.1. MR Analysis

We used the inverse-variance weighted (IVW) method as the main method analysis to assess the bidirectional causal associations between obesity-related traits, MASLD, and PLC in the discovery and replication datasets. To enhance the reliability of the findings, we combined the results of the two datasets through meta-analysis. We also used the weighted median to assess the robustness of our findings, which provides robust results even if the IVs are only 50% valid [34]. The sensitivity analysis of MR-Egger regression was used to detect and correct the horizontal pleiotropy of the IVs [35]. *p* values less than the Bonferroni-adjusted threshold of 0.007 (0.05/7 obesity-related traits) were considered statistically significant. *p* ≥ 0.007 and  ≤0.05 were regarded as suggestive associations.

The F value and R^2^ were calculated to evaluate the strength and interpretability of the IVs. We calculated the statistical power of the MR analysis using an online website (https://sb452.shinyapps.io/power/, accessed on 30 June 2025). Additionally, we assessed the heterogeneity of the IVs by Cochran’s Q test in order to test whether a single SNP contributed to the causal estimation. *p* < 0.1 in the Cochran’s Q test indicated heterogeneity. We also used the intercept of MR-Egger regression to assess the horizontal pleiotropy of the IVs. The outliers were identified by the MR–pleiotropy residual sum and outlier (MR-PRESSO) global test [36]. The same MR analysis was used to assess the causal associations between obesity-related traits and iron homeostasis biomarkers in the two iron homeostasis datasets, and the combined effects were calculated. Using two-sample MR analysis, we also investigated the causal effects of obesity-related traits on biomarkers of liver injury and hepatic steatosis.

#### 2.4.2. Mediation Analysis

Two-step MVMR analysis was performed to study the mediating effects of iron homeostasis biomarkers linking obesity with MASLD and PLC. The IVW method was the primary method utilized in the discovery stage to evaluate the causal effects of obesity-related traits on iron homeostasis biomarkers (β1) in iron homeostasis dataset 1. MVMR adjusted for genetically predicted obesity-related traits was used to assess the independent effects of iron homeostasis biomarkers on two MASLD and PLC datasets, respectively. We calculated MVMR instrument validity and MVMR directional pleiotropy to assess pleiotropy. The combined effects of the IVW method in the two MASLD and PLC datasets were pooled by common effect and random effects meta-analysis (combined_β2). When the result of the test for the heterogeneity of the meta-analysis was less than 0.05 or I^2^ was greater than 50%, random effects models were adopted, and vice versa. The mediation effects were calculated using the product of coefficients method: β1 × combined_β2. The total effects were the combined effects of the IVW method on obesity-related traits and MASLD or PLC (combined_β3). Thus, the proportion of the total effect mediated by iron homeostasis was estimated by dividing the indirect effect by the total effect: β1 × combined_β2/combined_β3. The standard error of the mediation estimate was calculated using the product distribution test [37]. *p* values less than the Bonferroni-adjusted threshold of 0.017 (0.05/3 iron homeostasis indicators) were considered statistically significant. To validate our results, we conducted the same mediation analysis between obesity, iron homeostasis biomarkers, MASLD, and PLC in iron homeostasis dataset 2.

All analyses were performed using the R packages “TwoSampleMR” [33], “MendelianRandomization [38]”, “MRPRESSO [36]”, and “Rmediation [37]” in R v.4.5.1 (https://www.r-project.org/).

## 3. Results

### 3.1. Causal Estimation from Obesity-Related Traits to MASLD and PLC Risks

**Figure 2:**
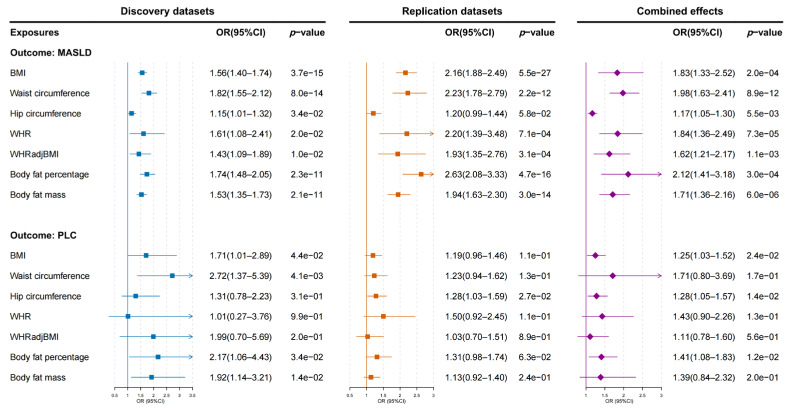
The causal effects of obesity-related traits on the risks of MASLD and PLC in the discovery datasets, replication datasets, and their combined effects. *p* values less than the Bonferroni-adjusted threshold of 0.007 (0.05/7) were considered statistically significant. OR: odds ratio; CI: confidence interval; BMI: body mass index; WHR: waist–hip ratio; WHRadjBMI: WHR adjusted for BMI; MASLD: metabolic dysfunction-associated steatotic liver disease; PLC: primary liver cancer.

We first assessed the causal relationships between obesity-related traits, MASLD, and PLC risks in the discovery and replication datasets (Tables S3 and S4). The IVW method showed that body fat percentage, body fat mass, BMI, waist circumference, and WHRadjBMI had consistent causal effects on MASLD risk in both datasets. After conducting a meta-analysis, we discovered that all obesity-related traits causally increased the probability of MASLD (Figure 2). Among these factors, body fat percentage presented the strongest risk effects, doubling the risk of MASLD (OR: 2.12; 95% CI: 1.41–3.18; *p* = 3.00 × 10^−4^). Waist circumference had stronger risk effects on MASLD (OR: 1.98; 95% CI: 1.63–2.41; *p* = 8.89 × 10^−12^) than BMI (OR: 1.83; 95% CI: 1.33–2.52; *p* = 2.04 × 10^−4^). Moreover, we found that waist circumference displayed significant causal effects on PLC risk in the discovery datasets (OR: 2.72; 95% CI: 1.37–5.39; *p* = 4.08 × 10^−3^). However, owing to significant heterogeneity (Phet = 0.034), no significant causal effects of waist circumference on PLC risk were observed in the pooled datasets. Genetically predicted BMI, hip circumference, and body fat percentage were marginally statistically linked to increased PLC risk in the combined analysis (*p* ≤ 0.05). The overall causal effects were directionally consistent with the sensitivity analysis performed via the weighted median and MR-Egger regression methods. Additionally, we investigated the causal associations between obesity-related traits and biomarkers of liver injury and hepatic steatosis. The results showed that genetic liability to BMI, waist circumference, and WHRadjBMI were significantly associated with ALT, AST, GGT, ALP, triglycerides, and fasting blood insulin levels after Bonferroni correction (Supplementary Data S17).

In the reverse MR analysis, genetically predicted risks for MASLD and PLC were not substantially correlated with any obesity-related traits (Tables S5 and S6). The strengths of the IVs and the statistical power of all the analyses are displayed in Table S7. The F values for all the IVs were above the threshold of 10 in both the discovery and replication datasets, ranging from 47 to 66, indicating that the IVs had enough power and representativeness for the exposures. In the causal estimates of MASLD, the statistical power was greater than 80% for all variables except hip circumference, indicating that the power was sufficient to detect the observed ORs. However, owing to the limited sample sizes of PLC cases, the statistical power was low in the analysis of PLC, especially in the replication datasets. In addition, we noted high heterogeneity in the MASLD analysis (Table S8), so we used multiplicative random effects in the primary IVW analysis. Except for WHRadjBMI, there was no indication of any bias in the causal estimation from obesity-related traits to MASLD and PLC risks caused by pleiotropy and outliers (*p* > 0.05).

### 3.2. Causal Estimation from Obesity-Related Traits to Iron Homeostasis Biomarkers

**Figure 3:**
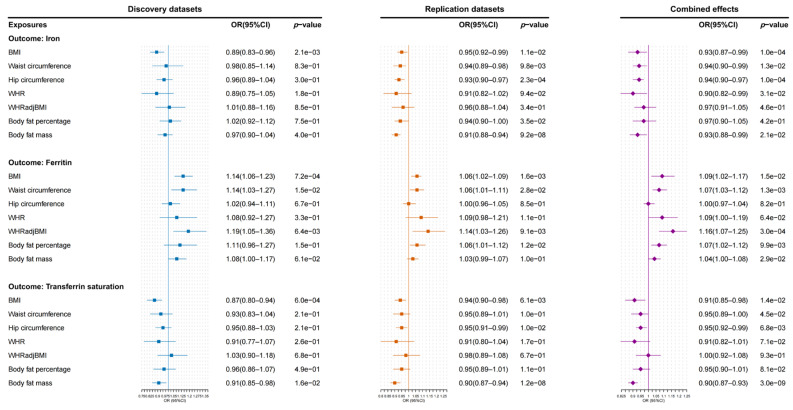
The causal effects of obesity-related traits on iron homeostasis biomarkers in the discovery datasets, replication datasets, and their combined effects. *p* values less than the Bonferroni-adjusted threshold of 0.007 (0.05/7) were considered statistically significant. OR: odds ratio; CI: confidence interval; BMI: body mass index; WHR: waist–hip ratio; WHRadjBMI: WHR adjusted for BMI.

Using two-sample MR analysis, we investigated the causal relationships between obesity-related traits and iron homeostasis biomarkers (Figure 3). In both of the iron homeostasis datasets, the effect directions of obesity-related traits on iron, ferritin, and transferrin saturation were consistent. In the combined effects, we discovered that, per 1 SD genetically predicted increased BMI was causally associated with reduced serum iron (OR: 0.93; 95% CI: 0.87–0.99; *p* = 1.00 × 10^−4^) and transferrin saturation (OR: 0.91; 95% CI: 0.85–0.98; *p* = 1.44 × 10^−2^), but increased ferritin (OR: 1.09; 95% CI: 1.02–117; *p* = 1.46 × 10^−2^). Additionally, we found that waist circumference (OR: 1.07; 95% CI: 1.03–1.12; *p* = 1.30 × 10^−3^) and WHRadjBMI (OR: 1.16; 95% CI: 1.07–1.25; *p* = 3.00 × 10^−4^) were both causally associated with elevated ferritin levels in terms of the combined effects. The causal effects were directionally consistent with the results of the weighted median and MR-Egger regression methods (Table S9). No horizontal pleiotropy was observed in these analyses (Table S10).

### 3.3. Causal Estimation from Iron Homeostasis Biomarkers to MASLD and PLC Risks

**Figure 4:**
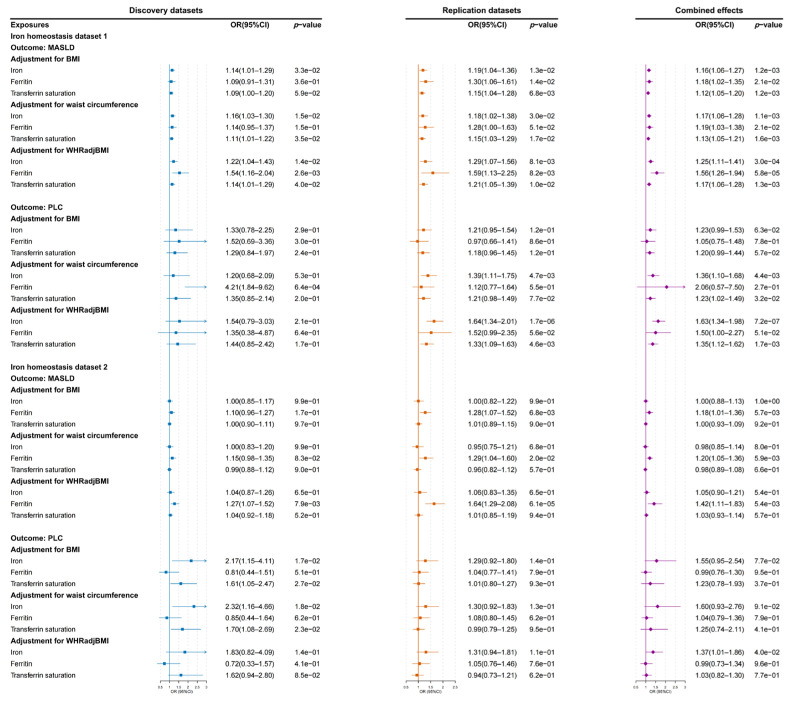
The causal effects of iron homeostasis biomarkers on the risks of MASLD and PLC after adjustment for BMI, waist circumference, and WHRadjBMI in the two iron homeostasis datasets. *p* values less than the Bonferroni-adjusted threshold of 0.017 (0.05/3) were considered statistically significant. OR: odds ratio; CI: confidence interval; BMI: body mass index; WHRadjBMI: waist–hip ratio adjusted for BMI; MASLD: metabolic dysfunction-associated steatotic liver disease; PLC: primary liver cancer.

Using MVMR analysis, we further investigated the causal associations between iron homeostasis biomarkers and MASLD or PLC risks after adjusting for obesity-related traits in two iron homeostasis datasets (Figure 4 and Table S11). In iron homeostasis dataset 1, we found that each 1 SD unit of higher serum iron (OR: 1.16; 95% CI: 1.06–1.27; *p* = 1.20 × 10^−3^), ferritin (OR: 1.18; 95% CI: 1.02–1.35; *p* = 2.10 × 10^−2^), and transferrin saturation (OR: 1.12; 95% CI: 1.05–1.20; *p* = 1.20 × 10^−3^) was associated with increased risks of MASLD after adjusting for BMI. The associations between ferritin and MASLD risk became stronger with adjustment for genetically predicted WHRadjBMI in the combined effects of iron homeostasis dataset 1 (OR: 1.56; 95% CI: 1.26–1.94; *p* = 5.83 × 10^−5^). The causal effects of ferritin on MASLD risk were successfully replicated in iron homeostasis dataset 2 after adjusting for obesity-related traits.

No significant associations were observed between iron homeostasis and PLC risk after adjusting for BMI in both iron homeostasis datasets. After correcting for WHRadjBMI, serum iron was significantly associated with PLC risk in iron homeostasis dataset 1 (OR: 1.63; 95% CI: 1.34–1.98; *p* = 7.18 × 10^−7^) and iron homeostasis dataset 2 (OR: 1.37; 95% CI: 1.01–1.86; *p* = 4.01 × 10^−2^). However, owing to the high heterogeneity, we only observed that a genetic predisposition toward increased ferritin increased the risk of PLC after correcting for waist circumference in the discovery stage of iron homeostasis dataset 1 (OR: 4.21; 95% CI: 1.84–9.62; *p* = 6.43 × 10^−4^).

### 3.4. Mediation Analysis

**Figure 5:**
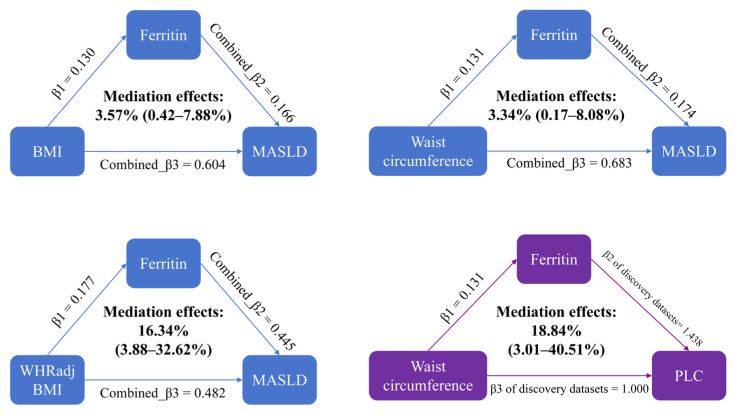
The mediation effects of ferritin linking obesity with MASLD and PLC in iron homeostasis dataset 1. β1: The IVW results of the associations between obesity-related traits and iron homeostasis biomarkers; Combined_β2: the pooled results of the associations between iron homeostasis biomarkers, MASLD, and PLC after adjustment for obesity-related traits; Combined_β3: the combined effects of IVW on obesity-related traits, MASLD, and PLC. BMI: body mass index; WHRadjBMI: waist–hip ratio adjusted for BMI; MASLD: metabolic dysfunction-associated steatotic liver disease; PLC: primary liver cancer.

In both iron homeostasis datasets, BMI, waist circumference, and WHRadjBMI were positively associated with ferritin levels (Figure 3). Ferritin was also linked to higher risks of MASLD and PLC after adjusting for obesity-related traits (Figure 4). We conducted a two-step MR analysis to explore the mediating effects of ferritin linking obesity with MASLD and PLC (Figure 5). We first explored the causal pathways from genetically predicted BMI, waist circumference, and WHRadjBMI to MASLD risk in iron homeostasis dataset 1. We found that the mediation effects of genetically predicted ferritin accounted for only approximately 3% from BMI and waist circumference to MASLD. However, genetic liability to ferritin mediated 16.34% (95% CI: 3.88–32.62%) of the effects of WHRadjBMI on MASLD risk. Moreover, in the causal pathway from genetically predicted waist circumference to PLC, the mediation effect of genetically predicted ferritin accounted for 18.84% (95% CI: 3.01–40.51%). The mediation effects of ferritin linking BMI, waist circumference, and WHRadjBMI with MASLD risk were consistent in iron homeostasis dataset 2, which strengthened our confidence in this causal inference (Figure S1).

## 4. Discussion

By two-sample bidirectional MR analysis, our study revealed that obesity-related traits causally increased the likelihood of MASLD. Body fat percentage had the most significant risk effects, while waist circumference demonstrated greater risk effects on MASLD risk compared to BMI. Moreover, we observed that a genetic predisposition to greater waist circumference, but not BMI, increased the risk of PLC. In the reverse MR analysis, we did not observe associations between genetically predicted obesity-related traits and MASLD or PLC risks.

These results are consistent with those of observational studies. Parente et al. [39], reported that waist-to-height ratio had greater diagnostic value than BMI for NAFLD patients with type 1 diabetes, and screening for waist-to-height ratio was recommended in this population. Machado et al. [40], revealed that relative-fat-mass-defined obesity better predicted cardiovascular metabolic disorders. Overall, these results suggest that body fat percentage plays an important role in the development of MASLD, even better than BMI, which is currently the most widely used but also the most controversial measurement [41]. Interestingly, our study also revealed that waist circumference had stronger causal effects on PLC risk in the discovery datasets compared with BMI (ORs were 2.72 for waist circumference and 1.71 for BMI), suggesting that waist circumference was a superior predictor of PLC risk. These results suggest that reducing waist circumference may be beneficial for people with MASLD and PLC [42].

Observational studies might be interfered with by confounding factors and reverse causality. For instance, we cannot know whether increased waist circumference was an upstream cause or a downstream consequence of MASLD or PLC. Therefore, it is necessary to use MR analysis to infer the causation between obesity-related traits, MASLD, and PLC risks. Recently, Xu et al. [43] employed MR analysis and revealed that liver fat and visceral adipose tissue distributions were more strongly linked to increased PLC risk than BMI; this finding was further corroborated by our study. Thus, we deemed that central obesity might be a more promising predictor and management indicator for patients with MASLD or PLC than general obesity. Although the relationships among obesity, MAFLD, and PLC have been extensively studied, the mechanisms involved are not fully understood. We previously conducted an MR study and reported that liver iron levels were associated with increased risks of MASLD [20]. Therefore, we performed a mediated MR analysis to explore the mediating effects of iron homeostasis linking obesity with MASLD and PLC.

Our results showed that genetically predicted increased BMI was significantly associated with reduced serum iron and transferrin saturation but increased ferritin levels, which was consistent with the findings of observational and MR studies [44]. We also found that other obesity-related traits were associated with increased ferritin and decreased transferrin saturation. The possible mechanisms for this involve obesity decreasing iron absorption and release from storage through increasing hepcidin levels, and increasing ferritin levels through mediating inflammation [45]. After adjusting for obesity-related traits, we found that serum iron, ferritin, and transferrin saturation were associated with increased risks of MASLD in both iron homeostasis datasets. Additionally, in iron homeostasis dataset 1, ferritin mediated the largest effects of WHRadjBMI on MASLD risk (16.34%; 95% CI: 3.88–32.62%). The mediation effects were further validated in iron homeostasis dataset 2, where the effects of WHRadjBMI on MASLD risk accounted for 9.54% (95% CI: 1.33–21.29%). Therefore, we concluded that obesity increased the risk of MASLD, which was partially mediated by increasing ferritin levels. Similarly, we found that ferritin was associated with an increased risk of PLC after correcting for waist circumference in the discovery datasets. However, owing to the large heterogeneity between the two PLC datasets, conclusions need to be drawn with some caution. We also observed that serum iron was significantly associated with PLC risk after correcting for WHRadjBMI, which could be explained by the recent study which reported that mitochondrial iron accumulation promoted lipid ROS generation and was involved in the development and cancer therapy of HCC [46]. Overall, our study established a relationship between iron homeostatic imbalance and the risks of MASLD or PLC, but the specific mechanisms remain to be further elucidated.

Our study is the first to explore the mediating effects of iron homeostasis biomarkers linking obesity with MASLD and PLC risks. To increase the robustness and validity of the findings, we performed discovery and replication analyses and combined the results of the two datasets through meta-analysis. The statistical power for MASLD was more than 80%, suggesting sufficient power to detect the observed ORs. Sensitivity analyses, including the WM and MR-Egger regression methods, also support the robustness of our causal estimates. However, there was limited statistical power for PLC in our analysis; furthermore, large-scale population studies are needed.

One of the limitations of our study was its representativeness and extrapolation. The populations included in our analysis were European on the basis of the public databases; therefore, other populations also need to be further studied. Moreover, research found that fat deposition patterns were correlated with a heightened prevalence of NAFLD in both genders, but their impact on fibrosis was exclusively evident in females [47], suggesting that sexual dimorphism in fat distribution may affect the advancement of liver disorders. Consequently, future studies must consider gender and investigate the influence of various gender subgroups on our research findings. Additionally, the pleiotropy of MR analysis needs to be emphasized [36]. We did not observe significant horizontal pleiotropy in the causal estimation by MR-Egger regression, but we could not exclude the existence of pleiotropic effects completely. Although we used MVMR correction for the effects of obesity-related traits, other potential confounding factors need to be considered as well. In addition, we were unable to estimate sample overlap between the datasets in this study. However, we conducted a two-sample MR analysis, and the F values for all the IVs were above the threshold of 10 in both the discovery and replication datasets, indicating that the IVs had enough explanatory power and representativeness for the exposures and minimized the effects of sample overlap.

## 5. Conclusions

Taken together, the results of our study reveal the stronger causal effects of waist circumference on MASLD and PLC risks compared to BMI. From genetically predicted waist circumference to MASLD and PLC risks, ferritin acted as a partial mediator. These results contribute to the growing body of evidence linking central obesity, iron homeostasis biomarkers, and liver diseases. Our studies emphasize the importance of improving central obesity in the management of patients with MASLD and PLC. Additional studies are needed to elucidate the specific mechanisms by which central obesity and iron homeostasis affect MASLD and PLC risks.

## Data Availability

The datasets supporting the conclusions of this article are available in the public database of the MR-Base platform (https://www.mrbase.org, accessed on 30 June 2025). Summary-level data about iron homeostasis biomarkers are available from https://www.decode.com/summarydata/ (accessed on 30 June 2025). We used R v.4.5.1 to generate and analyze the data. All codes are available from https://github.com/zypjyk/MR-analysis (accessed on 30 June 2025).

## Supplementary Materials

The following supporting information can be downloaded at: https://www.mdpi.com/article/10.3390/biomedicines13071641/s1, Figure S1: Mediation effects of ferritin linking obesity with MASLD in the iron homeostasis dataset 2.; Table S1: Characteristics of the continuous phenotypes; Table S2. Characteristics of the binary phenotypes; Table S3. Causal effects of obesity-related traits and the risk of MASLD in the discovery datasets, replication datasets and the combined effects; Table S4. Causal effects of obesity-related traits and the risk of PLC in the discovery datasets, replication datasets and the combined effects; Table S5. Causal effects of MASLD and obesity-related traits in the discovery datasets and replication datasets; Table S6. Causal effects of PLC and obesity-related traits in the discovery datasets and replication datasets; Table S7. Power calculation for the causal estimate from obesity-related traits to the risks of MASLD and PLC; Table S8. Heterogeneity and horizontal pleiotropic of the associations between obesity-related traits and the risks of MASLD and PLC; Table S9. Causal effects of obesity-related traits and iron homeostasis; Table S10. Heterogeneity and horizontal pleiotropy of the associations between obesity-related traits and iron homeostasis biomarkers; Table S11. The causal association between iron homeostasis and MASLD or PLC with adjustment for genetically predicted BMI, waist circumference and WHRadjBMI; Data S1. SNPs utilized as instrumental variables related to BMI; Data S2. SNPs utilized as instrumental variables related to waist circumference; Data S3. SNPs utilized as instrumental variables related to hip circumference; Data S4. SNPs utilized as instrumental variables related to waist-to-hip ratio; Data S5. SNPs utilized as instrumental variables related to WHRadjBMI; Data S6. SNPs utilized as instrumental variables related to body fat percentage; Data S7. SNPs utilized as instrumental variables related to whole body fat mass; Data S8. SNPs utilized as instrumental variables related to iron and BMI in multivariable MR analysis; Data S9. SNPs utilized as instrumental variables related to ferritin and BMI in multivariable MR analysis; Data S10. SNPs utilized as instrumental variables related to transferrin saturation and BMI in multivariable MR analysis; Data S11. SNPs utilized as instrumental variables related to iron and waist circumference in multivariable MR analysis; Data S12. SNPs utilized as instrumental variables related to ferritin and waist circumference in multivariable MR analysis; Data S13. SNPs utilized as instrumental variables related to transferrin saturation and waist circumference in multivariable MR analysis; Data S14. SNPs utilized as instrumental variables related to iron and WHRadjBMI in multivariable MR analysis; Data S15. SNPs utilized as instrumental variables related to ferritin and WHRadjBMI in multivariable MR analysis; Data S16. SNPs utilized as instrumental variables related to transferrin saturation and WHRadjBMI in multivariable MR analysis; Data S17. Causal estimate from obesity-related traits to biomarkers of hepatic steatosis and liver injury.
