# Supplementary material for: Iron Homeostasis as a Mediator Linking Central Obesity with MASLD and Primary Liver Cancer: A Two-Step Mendelian Randomization Study
Source: Biomedicines. 2025 Jul 4;13(7):1641. doi: 10.3390/biomedicines13071641 (PMC12292972; doi:10.3390/biomedicines13071641)
Supplement: Supplementary file 1 [file biomedicines-13-01641-s001.zip › Supplementary Tables and Figures.pdf]

Table S1. Characteristics of the continuous phenotypes.

Phenotypes	*Authors*	*Consortium/PMID*	*Sample sizes*
**Obesity-related traits**			
BMI	Neale	Neale Lab	336107
Waist circumference	Neale	Neale Lab	336639
Hip circumference	Neale	Neale Lab	336601
WHR	Shungin D	GIANT/25673412	212244
WHRadjBMI	Shungin D	GIANT/25673412	210082
Body fat percentage	Ben Elsworth	MRC-IEU	454633
Body fat mass	Ben Elsworth	MRC-IEU	454137
**Iron homeostasis**			
Iron	Benyamin	GIS/25352340	23986
Ferritin	Benyamin	GIS/25352340	23986
Transferrin saturation	Benyamin	GIS/25352340	23986
Iron	Bell S	33536631	163511
Ferritin	Bell S	33536631	246139
Transferrin saturation	Bell S	33536631	131471
**Biomarkers of liver injury and hepatic steatosis**			
Alanine aminotransferase (ALT)	Sakaue S	34594039	344136
Aspartate aminotransferase (AST)	Sakaue S	34594039	342990
Gamma glutamyl transpeptidase (GGT)	Sakaue S	34594039	344104
Alkaline phosphatase (ALP)	Sakaue S	34594039	344292
Triglycerides (TG)	Sakaue S	34594039	343992
Fasting blood insulin	Manning AK	22581228	51750

BMI: body mass index; WHR: waist-hip ratio; WHRadjBMI: WHR adjusted for BMI.

Table S2. Characteristics of the binary phenotypes.

Phenotypes	*Authors*	*Consortium/PMID*	*Sample sizes*	*Case/Control*
MASLD	Ghodsian N	34841290	778614	8434/770180
MASLD	Fairfield CJ	34535985	377998	4761/373227
PLC	-	FinnGen	218792	304/218488
PLC	Sakaue S	34594039	475638	379/475259

MASLD: metabolic dysfunction-associated steatotic liver disease; PLC: primary liver cancer.

Table S3. Causal effects of obesity-related traits and the risk of MASLD in the discovery datasets, replication datasets and the combined effects.

Exposures	*Inverse-variance weighted*		*Weighted median*		*MR-Egger regression*	
	OR (95%CI)	*P*	*OR (95%CI)*	*P*	*OR (95%CI)*	*P*
**Discovery datasets**						
BMI	1.56(1.40-1.74)	3.70E-15	1.58(1.33-1.87)	1.71E-07	1.72(1.25-2.37)	9.96E-04
Waist circumference	1.82(1.55-2.12)	8.01E-14	1.85(1.50-2.28)	1.14E-08	1.86(1.15-3.01)	1.19E-02
Hip circumference	1.15(1.01-1.32)	3.43E-02	1.15(0.97-1.37)	1.15E-01	0.99(0.68-1.44	9.46E-01
WHR	1.61(1.08-2.41)	2.03E-02	1.33(0.86-2.04)	1.98E-01	1.90(0.29-12.33)	5.05E-01
WHRadjBMI	1.43(1.09-1.89)	1.01E-02	1.67(1.21-2.31)	1.91E-03	2.58(0.70-9.54)	1.64E-01
Body fat percentage	1.74(1.48-2.05)	2.26E-11	1.79(1.44-2.22)	1.78E-07	2.43(1.44-4.09)	9.40E-04
Body fat mass	1.53(1.35-1.73)	2.07E-11	1.61(1.37-1.89)	5.48E-09	1.70(1.21-2.41)	2.71E-03
**Replication datasets**						
BMI	2.16(1.88-2.49)	5.53E-27	2.15(1.70-2.71)	1.12E-10	1.54(1.02-2.32)	3.89E-02
Waist circumference	2.23(1.78-2.79)	2.24E-12	2.34(1.74-3.14)	2.12E-08	1.35(0.68-2.69)	3.96E-01
Hip circumference	1.20(0.99-1.44)	5.77E-02	1.38(1.10-1.73)	5.14E-03	0.83(0.48-1.41)	4.91E-01
WHR	2.20(1.39-3.48)	7.10E-04	3.09(1.83-5.23)	2.55E-05	3.28(0.39-27.90)	2.86E-01

WHRadjBMI	1.93(1.35-2.76)	3.14E-04	2.17(1.44-3.27)	2.15E-04	10.69(2.09-54.58)	7.41E-03
Body fat percentage	2.63(2.08-3.33)	4.69E-16	2.40(1.77-3.25)	1.42E-08	1.42(0.67-3.00)	3.57E-01
Body fat mass	1.94(1.63-2.30)	3.03E-14	1.90(1.52-2.39)	2.52E-08	1.32(0.82-2.12)	2.55E-01
**Combined effects**						
BMI	1.83(1.33-2.52)	2.04E-04	1.82(1.35-2.46)	9.24E-05	1.65(1.28-2.12)	1.01E-04
Waist circumference	1.98(1.63-2.41)	8.89E-12	2.03(1.62-2.54)	7.18E-10	1.67(1.13-2.48)	1.04E-02
Hip circumference	1.17(1.05-1.30)	5.53E-03	1.24(1.04-1.48)	1.64E-02	0.93(0.69-1.27)	6.67E-01
WHR	1.84(1.36-2.49)	7.25E-05	2.00(0.88-4.57)	1.00E-01	2.41(0.59-9.86)	2.21E-01
WHRadjBMI	1.62(1.21-2.17)	1.07E-03	1.85(1.43-2.38)	2.20E-06	4.81(1.21-19.17)	2.59E-02
Body fat percentage	2.12(1.41-3.18)	3.00E-04	2.03(1.53-2.70)	1.06E-06	2.04(1.33-3.13)	1.10E-03
Body fat mass	1.71(1.36-2.16)	6.00E-06	1.70(1.49-1.94)	1.87E-15	1.56(1.18-2.06)	1.80E-03

OR: odds ratio; CI: confidence interval; BMI: body mass index; WHR: waist-hip ratio; WHRadjBMI: WHR adjusted for BMI; MASLD: metabolic dysfunction-associated steatotic liver disease.

Table S4. Causal effects of obesity-related traits and the risk of PLC in the discovery datasets, replication datasets and the combined effects.

Exposures	*Inverse-variance weighted*		*Weighted median*		*MR-Egger regression*	
	OR (95%CI)	*P*	*OR (95%CI)*	*P*	*OR (95%CI)*	*P*
**Discovery datasets**						
BMI	1.71(1.01-2.89)	4.43E-02	2.13(0.88-5.15)	9.37E-02	3.48(0.74-16.37)	1.15E-01
Waist circumference	2.72(1.37-5.39)	4.08E-03	3.12(1.01-9.68)	4.89E-02	5.73(0.69-47.68)	1.08E-01
Hip circumference	1.31(0.78-2.23)	3.11E-01	1.51(0.64-3.59)	3.46E-01	1.32(0.29-5.98)	7.18E-01
WHR	1.01(0.27-3.76)	9.94E-01	0.93(0.12-7.43)	9.47E-01	0.01(0.000-3.23)	1.28E-01
WHRadjBMI	1.99(0.70-5.69)	1.98E-01	1.20(0.25-5.87)	8.19E-01	0.01(0.000-1.07)	6.14E-02
Body fat percentage	2.17(1.06-4.43)	3.44E-02	1.61(0.51-5.08)	4.15E-01	2.98(0.29-30.24)	3.57E-01
Body fat mass	1.92(1.14-3.21)	1.37E-02	2.14(0.92-4.98)	7.85E-02	1.37(0.32-5.88)	6.68E-01
**Replication datasets**						
BMI	1.19(0.96-1.46)	1.05E-01	1.00(0.71-1.41)	9.94E-01	1.26(0.68-2.32)	4.68E-01
Waist circumference	1.23(0.94-1.62)	1.33E-01	1.00(0.64-1.56)	9.83E-01	1.31(0.55-3.12)	5.46E-01
Hip circumference	1.28(1.03-1.59)	2.65E-02	0.99(0.69-1.41)	9.49E-01	0.80(0.40-1.61)	5.32E-01
WHR	1.50(0.92-2.45)	1.06E-01	1.33(0.68-2.60)	3.97E-01	0.80(0.09-6.86)	8.41E-01
WHRadjBMI	1.03(0.70-1.51)	8.86E-01	1.15(0.67-1.98)	6.19E-01	1.47(0.24-8.95)	6.78E-01
Body fat percentage	1.31(0.98-1.74)	6.35E-02	1.18(0.74-1.88)	4.91E-01	1.16(0.46-2.93)	7.48E-01
Body fat mass	1.13(0.92-1.40	2.42E-01	0.88(0.62-1.23)	4.40E-01	0.88(0.48-1.60)	6.70E-01
**Combined effects**						

BMI	1.25(1.03-1.52)	2.43E-02	1.30(0.64-2.64)	4.64E-01	1.45(0.82-2.56)	2.05E-01
Waist circumference	1.71(0.80-3.69)	1.69E-01	1.56(0.53-4.64)	4.22E-01	1.63(0.73-3.62)	2.39E-01
Hip circumference	1.28(1.05-1.57)	1.45E-02	1.05(0.76-1.46)	7.58E-01	0.87(0.46-1.64)	6.74E-01
WHR	1.43(0.90-2.26)	1.27E-01	1.29(0.68-2.43)	4.41E-01	0.80(0.09-6.98)	8.40E-01
WHRadjBMI	1.11(0.78-1.60)	5.59E-01	1.15(0.69-1.93)	5.81E-01	1.47(0.24-8.98)	6.76E-01
Body fat percentage	1.41(1.08-1.83)	1.23E-02	1.23(0.80-1.90)	3.42E-01	1.32(0.56-3.12)	5.27E-01
Body fat mass	1.39(0.84-2.32)	2.00E-01	1.26(0.54-2.95)	5.99E-01	0.94(0.54-1.64)	8.24E-01

OR: odds ratio; CI: confidence interval; BMI: body mass index; WHR: waist-hip ratio; WHRadjBMI: WHR adjusted for BMI; PLC: primary liver cancer.

Table S5. Causal effects of MASLD and obesity-related traits in the discovery datasets and replication datasets.

Outcomes	*Inverse-variance weighted*		*Weighted median*		*MR-Egger regression*	
	OR (95%CI)	***P***	***OR (95%CI)***	***P***	***OR (95%CI)***	***P***
**Discovery datasets**						
BMI	1 (0.93-1.07)	0.991	0.98 (0.96-1.00)	0.059	0.88(0.75-1.04)	0.268
Waist circumference	1.01(0.95-1.08)	0.678	1.00(0.98-1.01)	0.637	0.92(0.77-1.11)	0.475
Hip circumference	0.98(0.94-1.02)	0.325	0.97(0.96-0.99)	0.003	0.92(0.82-1.05)	0.340
WHR	1.03 (0.99-1.07)	0.205				
WHRadjBMI	1.05 (1.01-1.10)	0.013				
Body fat percentage	1.00(0.94-1.05)	0.910	0.99(0.98-1.00)	0.045	0.99(0.85-1.15)	0.885
Body fat mass	0.98(0.91-1.06)	0.688	0.98(0.96-0.99)	0.005	0.99(0.80-1.22)	0.913
**Replication datasets**						
BMI	0.99(0.96-1.03)	0.725	0.98(0.96-0.99)	0.002	0.99(0.89-1.09)	0.776
Waist circumference	1.00(0.97-1.04)	0.821	1.00(0.98-1.01)	0.442	1.01(0.92-1.10)	0.871
Hip circumference	0.97(0.94-1.01)	0.129	0.98(0.97-1.00)	0.018	0.99(0.90-1.08)	0.755
WHR	1.02(1.00-1.04)	0.119	1.02(0.99-1.05)	0.154	1.03(0.93-1.14)	0.612
WHRadjBMI	1.04(1.00-1.08)	0.054	1.05(1.02-1.09)	0.001	1.06(0.94-1.20)	0.375
Body fat percentage	1.00(0.97-1.03)	0.869	0.99(0.98-1.00)	0.073	0.98(0.91-1.06)	0.663
Body fat mass	0.99(0.95-1.04)	0.738	0.98(0.97-0.99)	0.002	0.98(0.88-1.10)	0.777

OR: odds ratio; CI: confidence interval; BMI: body mass index; WHR: waist-hip ratio; WHRadjBMI: WHR adjusted for BMI; MASLD: metabolic dysfunction-associated steatotic liver disease.

Table S6. Causal effects of PLC and obesity-related traits in the discovery datasets and replication datasets.

Outcomes	***Inverse-variance weighted***		***Weighted median***		***MR-Egger regression***	
	OR (95%CI)	***P***	***OR (95%CI)***	***P***	***OR (95%CI)***	***P***
**Discovery datasets**						
BMI	1.00(0.97-1.02)	0.629	-	-	-	-
Waist circumference	1.00(0.99-1.01)	0.827	-	-	-	-
Hip circumference	1.00(0.98-1.02)	0.742	-	-	-	-
WHR	1.01(0.99-1.02)	0.597	-	-	-	-
WHRadjBMI	1.01(0.99-1.03)	0.367	-	-	-	-
Body fat percentage	1.00(0.99-1.00)	0.297	-	-	-	-
Body fat mass	1.00(0.98-1.01)	0.626	-	-	-	-
**Replication datasets**						
BMI	0.99(0.98-1.01)	0.437	1.00(0.99-1.01)	0.603	1.00(0.97-1.02)	0.854
Waist circumference	1.00(0.99-1.01)	0.921	1.00(0.99-1.01)	0.777	1.00(0.99-1.01)	0.746
Hip circumference	1.00(0.98-1.01)	0.583	0.99(0.99-1.00)	0.257	0.99(0.96-1.02)	0.548
WHR	1.03(1.00-1.06)	0.093	1.03(1.00-1.07)	0.071	0.97(0.52-1.80)	0.938
WHRadjBMI	1.03(0.99-1.06)	0.115	1.03(0.99-1.07)	0.184	0.81(0.46-1.41)	0.590
Body fat percentage	0.99(0.99-1.00)	0.072	1.00(0.99-1.00)	0.136	1.00(0.99-1.01)	0.647
Body fat mass	0.99(0.98-1.00)	0.277	1.00(0.99-1.00)	0.265	0.99(0.98-1.01)	0.519

OR: odds ratio; CI: confidence interval; BMI: body mass index; WHR: waist-hip ratio; WHRadjBMI: WHR adjusted for BMI; PLC: primary liver cancer.

Table S7. Power calculation for the causal estimate from obesity-related traits to the risks of MASLD and PLC.

Exposures	*Discovery datasets*					*Replication datasets*			
	SNPs	*R^2*	*F*	*OR*	*POWER*	*R^2*	*F*	*OR*	*POWER*
**Outcome: MASLD**									
BMI	309	4.93%	60	1.56	100%	5.1%	60	2.16	100%
Waist circumference	228	3.50%	57	1.82	100%	3.6%	56	2.23	100%
Hip circumference	282	4.71%	60	1.15	75.50%	4.8%	60	1.20	78.60%
WHR	29	0.61%	47	1.61	90.30%	0.6%	47	2.20	99.00%
WHRadjBMI	38	0.93%	56	1.43	85.30%	0.9%	56	1.93	99.30%
Body fat percentage	388	4.63%	61	1.74	100%	4.82%	62	2.63	100%
Body fat mass	430	5.53%	66	1.53	100%	5.70%	66	1.94	100%
**Outcome: PLC**									
BMI	309	4.95%	60	1.71	55.00%	5.01%	60	1.19	11.50%
Waist circumference	228	3.52%	56	2.72	90.70%	3.59%	56	1.23	11.60%
Hip circumference	282	4.72%	60	1.31	17.50%	4.66%	60	1.28	17.80%
WHR	29	0.61%	47	1.01	2.60%	0.61%	47	1.50	9.00%
WHRadjBMI	38	0.94%	56	1.99	21.40%	0.94%	56	1.03	2.80%
Body fat percentage	388	4.73%	62	2.17	83.80%	4.79%	61	1.31	21.00%
Body fat mass	430	5.60%	66	1.92	77.00%	5.61%	66	1.13	8.10%

SNPs: single nucleotide polymorphisms; R^2: the percentage of phenotypes explained by SNPs; F: the strength of correlation between SNPs and phenotypes; OR, odds ratio; BMI: body mass index; WHR: waist-hip ratio; WHRadjBMI: WHR adjusted for BMI; MASLD: metabolic dysfunction-associated steatotic liver disease; PLC: primary liver cancer.

Table S8. Heterogeneity and horizontal pleiotropic of the associations between obesity-related traits and the risks of MASLD and PLC.

Exposures	*Discovery datasets*					*Replication datasets*				
	Cochran's Q test		*MR- Egger regression*		*MR-PRESSO global test*	*Cochran's Q test*		*MR- Egger regression*		*MR-PRESSO global test*
	**Q-statistics**	***P***	**Intercept**	***P***		**Q-statistics**	***P***	**Intercept**	***P***	
**Outcome: MASLD**										
BMI	331.06	0.045	-0.002	0.517	0.050	356.13	0.012	0.007	0.085	0.735
Waist circumference	284.89	9.90E-04	0.000	0.913	0.764	383.86	8.99E-11	0.010	0.134	0.676
Hip circumference	432.49	3.14E-09	0.003	0.391	0.808	542.45	2.97E-19	0.008	0.153	0.683
WHR	62.84	1.11E-04	-0.004	0.859	0.964	49.89	0.005	-0.010	0.711	0.276
WHRadjBMI	57.49	0.007	-0.017	0.373	0.691	63.46	0.002	-0.050	0.043	0.518
Body fat percentage	470.09	7.06E-05	-0.005	0.191	0.866	650.85	2.20E-17	0.009	0.090	0.392
Body fat mass	590.02	2.71E-09	-0.002	0.519	0.727	732.40	6.61E-20	0.007	0.091	0.445
**Outcome: PLC**										
BMI	229.96	0.996	-0.015	0.340	0.997	296.48	0.449	-0.001	0.850	0.535
Waist circumference	198.58	0.797	-0.014	0.468	0.839	225.52	0.385	-0.001	0.888	0.427
Hip circumference	255.49	0.795	0.000	0.994	0.774	293.09	0.204	0.010	0.167	0.187
WHR	25.87	0.526	0.124	0.119	0.589	18.86	0.875	0.017	0.562	0.885
WHRadjBMI	40.53	0.239	0.166	0.031	0.180	23.05	0.940	-0.011	0.694	0.923
Body fat percentage	341.82	0.803	-0.005	0.778	0.830	361.20	0.619	0.002	0.793	0.701
Body fat mass	387.64	0.736	0.006	0.632	0.771	408.70	0.495	0.005	0.374	0.479

P<0.1 was considered significant pleiotropic effects. BMI: body mass index; WHR: waist-hip ratio; WHRadjBMI: WHR adjusted for BMI; MASLD: metabolic dysfunction-associated steatotic liver disease; PLC: primary liver cancer.

Table S9. Causal effects of obesity-related traits and iron homeostasis.

Exposures	*Outcomes*	*Inverse-variance weighted*		*Weighted median*		*MR-Egger regression*	
		OR (95%CI)	***P***	***OR (95%CI)***	***P***	***OR (95%CI)***	***P***
	iron	0.89(0.83-0.96)	0.002	0.92(0.81-1.05)	0.226	0.87(0.70-1.08)	0.205
BMI	Ferritin	1.14(1.06-1.23)	0.001	1.10(0.98-1.24)	0.091	1.02(0.82-1.27)	0.867
	Transferrin Saturation	0.87(0.80-0.94)	0.001	0.90(0.78-1.03)	0.121	0.82(0.65-1.03)	0.089
	iron	0.98(0.85-1.14)	0.830	0.93(0.75-1.16)	0.538	0.71(0.36-1.40)	0.325
Waist circumference	Ferritin	1.14(1.03-1.27)	0.015	1.00(0.86-1.17)	0.959	0.99(0.72-1.35)	0.931
	Transferrin Saturation	0.93(0.83-1.04)	0.208	0.87(0.73-1.04)	0.130	0.85(0.60-1.21)	0.367
	iron	0.96(0.89-1.04)	0.303	0.94(0.82-1.07)	0.342	0.89(0.70-1.14)	0.375
Hip circumference	Ferritin	1.02(0.94-1.11)	0.671	0.98(0.86-1.12)	0.790	1.08(0.83-1.41)	0.576
	Transferrin Saturation	0.95(0.88-1.03)	0.207	0.93(0.81-1.07)	0.300	0.85(0.66-1.09)	0.214
	iron	0.89(0.75-1.05)	0.180	0.90(0.71-1.16)	0.427	0.92(0.43-1.98)	0.837
WHR	Ferritin	1.08(0.92-1.27)	0.328	1.00(0.78-1.27)	0.969	0.75(0.33-1.68)	0.489
	Transferrin Saturation	0.91(0.77-1.07)	0.258	0.86(0.66-1.11)	0.237	0.88(0.35-2.17)	0.776
	iron	1.01(0.88-1.16)	0.854	1.10(0.90-1.35)	0.350	1.01(0.48-2.15)	0.969
WHRadjBMI	Ferritin	1.19(1.05-1.36)	0.006	1.27(1.05-1.53)	0.014	1.07(0.50-2.31)	0.862
	Transferrin Saturation	1.03(0.90-1.18)	0.685	1.09(0.88-1.34)	0.430	0.86(0.38-1.92)	0.711
Body fat percentage	iron	1.02(0.92-1.12)	0.755	0.99(0.84-1.18)	0.931	1.10(0.79-1.55)	0.571
	Ferritin	1.11(0.96-1.27)	0.148	1.13(0.93-1.38)	0.225	1.20(0.71-2.01)	0.494
	Transferrin Saturation	0.96(0.86-1.07)	0.486	0.89(0.76-1.05)	0.180	1.05(0.73-1.50)	0.804
Body fat mass	iron	0.97(0.90-1.04)	0.397	0.99(0.86-1.13)	0.834	0.96(0.77-1.20)	0.744
	Ferritin	1.08(1.00-1.17)	0.061	0.99(0.87-1.12)	0.873	1.00(0.78-1.27)	0.990
	Transferrin Saturation	0.91(0.85-0.98)	0.016	0.91(0.79-1.04)	0.178	0.91(0.73-1.14)	0.418

OR: odds ratio; CI: confidence interval; BMI: body mass index; WHR: waist-hip ratio; WHRadjBMI: WHR adjusted for BMI.

Table S10. Heterogeneity and horizontal pleiotropy of the associations between obesity-related traits and iron homeostasis biomarkers.

Exposures	*0utcomes*	*Cochran's Q test*		*MR- Egger regression*	
		**Q-statistics**	***P***	**Intercept**	***P***
BMI	iron	205.77	0.472	0.001	0.814
	Ferritin	243.74	0.033	0.002	0.293
	Transferrin Saturation	245.65	0.034	0.001	0.570
Waist circumference	iron	83.99	0.301	0.006	0.335
	Ferritin	184.31	0.017	0.003	0.342
	Transferrin Saturation	198.00	0.003	0.002	0.606
Hip circumference	iron	184.32	0.318	0.001	0.556
	Ferritin	221.07	0.007	-0.001	0.656
	Transferrin Saturation	192.52	0.201	0.002	0.370
WHR	iron	23.15	0.625	-0.001	0.930
	Ferritin	32.78	0.169	0.009	0.369
	Transferrin Saturation	34.94	0.113	0.001	0.937
WHRadjBMI	iron	36.02	0.329	0.000	0.996
	Ferritin	42.85	0.117	0.003	0.780
	Transferrin Saturation	41.87	0.139	0.005	0.655
Body fat percentage	iron	266.21	0.230	-0.001	0.619
	Ferritin	182.04	0.038	-0.001	0.757
	Transferrin Saturation	298.18	0.020	-0.001	0.628
Body fat mass	iron	287.10	0.240	0.000	0.965
	Ferritin	352.77	0.000	0.001	0.491
	Transferrin Saturation	295.79	0.154	0.000	0.993

BMI: body mass index; WHR: waist-hip ratio; WHRadjBMI: WHR adjusted for BMI.

Table S11. The causal association between iron homeostasis and MASLD or PLC with adjustment for genetically predicted BMI, waist circumference and WHRadjBMI.

Exposures	*0utcomes*	*Inverse-variance weighted*			*MR-Egger regression*			*MVMR Instrument validity*			*MVMR directional pleiotropy*	
		coef	*se*	*P*	*coef*	*se*	*P*	*F-statistic*	*Q statistic*	*P value*	*Egger*	*P*
Adjustment for BMI												
Iron	MASLD dataset1	0.131	0.061	0.033	0.179	0.069	0.009	26.91	241.23	0.097	-0.002	0.127
Ferritin	MASLD dataset1	0.086	0.094	0.357	0.263	0.121	0.029	24.80	246.91	0.061	-0.004	0.027
TS	MASLD dataset1	0.091	0.048	0.059	0.136	0.052	0.009	25.06	240.99	0.108	-0.003	0.027
Iron	MASLD dataset2	0.172	0.070	0.013	0.241	0.076	0.002	45.62	246.94	0.095	-0.004	0.033
Ferritin	MASLD dataset2	0.266	0.108	0.014	0.381	0.136	0.005	46.59	245.03	0.110	-0.003	0.170
TS	MASLD dataset2	0.141	0.052	0.007	0.181	0.055	0.001	46.59	245.80	0.112	-0.004	0.041
Adjustment for waist circumference												
Iron	MASLD dataset1	0.147	0.060	0.015	0.142	0.067	0.035	28.67	173.48	0.189	0.000	0.874
Ferritin	MASLD dataset1	0.134	0.094	0.154	0.379	0.118	0.001	26.74	181.53	0.128	-0.006	0.001
TS	MASLD dataset1	0.103	0.049	0.035	0.134	0.052	0.011	28.07	175.42	0.177	-0.003	0.121
Iron	MASLD dataset2	0.168	0.078	0.030	0.246	0.084	0.004	27.36	225.97	<0.001	-0.005	0.028
Ferritin	MASLD dataset2	0.243	0.125	0.051	0.386	0.155	0.013	25.95	233.19	<0.001	-0.004	0.133
TS	MASLD dataset2	0.142	0.059	0.017	0.196	0.062	0.001	28.95	225.82	0.001	-0.006	0.010
Adjustment for WHRadjBMI												
Iron	MASLD dataset1	0.199	0.081	0.014	0.176	0.090	0.051	5.38	54.85	0.007	0.003	0.521
Ferritin	MASLD dataset1	0.433	0.143	0.003	0.409	0.173	0.018	7.81	50.09	0.037	0.001	0.796
TS	MASLD dataset1	0.129	0.063	0.040	0.131	0.069	0.058	4.40	58.28	0.004	0.000	0.948
Iron	MASLD dataset2	0.257	0.097	0.008	0.221	0.106	0.037	9.17	64.00	0.001	0.005	0.387
Ferritin	MASLD dataset2	0.466	0.176	0.008	0.636	0.199	0.001	9.44	63.40	0.002	-0.011	0.092

TS	MASLD dataset2	0.187	0.073	0.010	0.165	0.078	0.035	8.86	65.28	0.001	0.005	0.410
Adjustment for BMI												
Iron	PLC dataset1	0.284	0.269	0.290	0.385	0.349	0.27	3.80	165.68	0.994	-0.004	0.547
Ferritin	PLC dataset1	0.417	0.405	0.304	0.617	0.623	0.321	3.90	163.65	0.996	-0.004	0.647
TS	PLC dataset1	0.255	0.216	0.238	0.207	0.271	0.445	3.62	166.91	0.994	0.003	0.688
Iron	PLC dataset2	0.192	0.123	0.120	0.301	0.141	0.032	1.68	219.22	0.445	-0.005	0.111
Ferritin	PLC dataset2	-0.035	0.193	0.856	0.04	0.269	0.882	0.40	214.31	0.539	-0.001	0.694
TS	PLC dataset2	0.163	0.106	0.122	0.227	0.118	0.055	1.49	218.21	0.483	-0.003	0.229
Adjustment for waist circumference												
Iron	PLC dataset1	0.178	0.286	0.533	0.466	0.346	0.178	4.73	139.77	0.835	-0.014	0.082
Ferritin	PLC dataset1	1.438	0.421	6.43E-04	0.995	0.620	0.109	10.46	129.76	0.962	0.010	0.275
TS	PLC dataset1	0.299	0.235	0.203	0.218	0.275	0.428	5.40	138.62	0.864	0.006	0.475
Iron	PLC dataset2	0.330	0.117	0.005	0.523	0.137	0.000	4.14	153.02	0.619	-0.009	0.005
Ferritin	PLC dataset2	0.117	0.193	0.545	0.341	0.264	0.197	0.39	163.20	0.459	-0.005	0.218
TS	PLC dataset2	0.190	0.107	0.077	0.284	0.121	0.019	1.70	160.22	0.480	-0.005	0.089
Adjustment for WHRadjBMI												
Iron	PLC dataset1	0.434	0.344	0.207	0.319	0.381	0.402	2.31	35.31	0.360	0.013	0.467
Ferritin	PLC dataset1	0.302	0.653	0.644	0.298	0.802	0.71	1.41	38.85	0.300	0.000	0.994
TS	PLC dataset1	0.363	0.267	0.173	0.266	0.29	0.359	2.52	35.27	0.408	0.015	0.381
Iron	PLC dataset2	0.494	0.103	1.73E-06	0.45	0.149	0.003	11.47	20.10	0.962	0.004	0.528
Ferritin	PLC dataset2	0.421	0.221	0.056	0.508	0.344	0.14	1.88	24.02	0.919	-0.003	0.688
TS	PLC dataset2	0.289	0.102	0.005	0.301	0.127	0.018	4.05	28.12	0.751	-0.001	0.828

TS: transferrin saturation; BMI: body mass index; WHR: waist-hip ratio; WHRadjBMI: WHR adjusted for BMI; MASLD: metabolic dysfunction-associated steatotic liver disease; PLC: primary liver cancer.

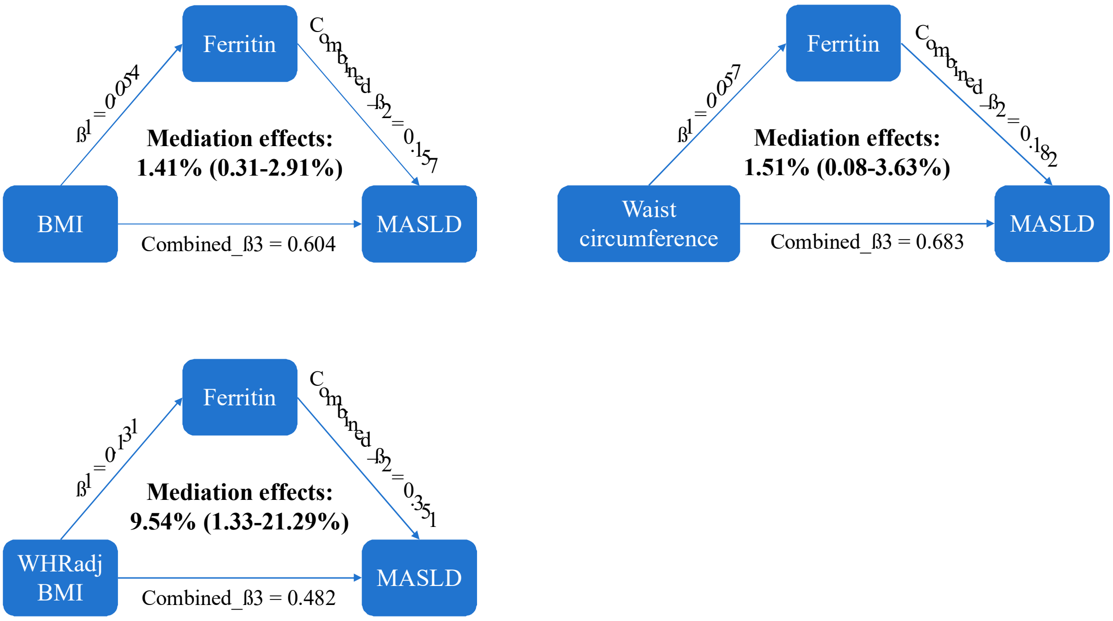

Figure S1: Mediation effects of ferritin linking obesity with MASLD in the iron homeostasis dataset 2. β 1: the IVW results of the association between obesity-related traits and iron homeostasis biomarkers; Combined_ β 2: the pooled results of the association between iron homeostasis biomarkers and MASLD after adjustment for obesity-related traits; Combined_ β 3: the combined effects of IVW from obesity-related traits to MASLD. BMI: body mass index; WHRadjBMI: waist-hip ratio adjusted for BMI; MASLD: metabolic dysfunction-associated steatotic liver disease.
